# VersaCount: customizable manual tally software for cell counting

**DOI:** 10.1186/1751-0473-5-1

**Published:** 2010-01-13

**Authors:** Charles C Kim, Joseph L DeRisi

**Affiliations:** 1 Department of Biochemistry and Biophysics, University of California San Francisco, San Francisco, CA 94158, USA

## Abstract

**Background:**

The manual counting of cells by microscopy is a commonly used technique across biological disciplines. Traditionally, hand tally counters have been used to track event counts. Although this method is adequate, there are a number of inefficiencies which arise when managing large numbers of samples or large sample sizes.

**Results:**

We describe software that mimics a traditional multi-register tally counter. Full customizability allows operation on any computer with minimal hardware requirements. The efficiency of counting large numbers of samples and/or large sample sizes is improved through the use of a "multi-count" register that allows single keystrokes to correspond to multiple events. Automatically updated multi-parameter values are implemented as user-specified equations, reducing errors and time required for manual calculations. The user interface was optimized for use with a touch screen and numeric keypad, eliminating the need for a full keyboard and mouse.

**Conclusions:**

Our software provides an inexpensive, flexible, and productivity-enhancing alternative to manual hand tally counters.

## Background

Cell counting is a technique used across many biological disciplines. For example, counting cell density on a hemocytometer is routinely used during *in vitro *cell culture and processing of tissue samples into single-cell suspensions. In another application, leukocyte and erythrocyte counts and morphology are commonly monitored in both clinical and basic research labs by microscopic examination of blood smeared onto slides. In our laboratory, dozens to hundreds of these smears are analyzed weekly in order monitor malaria parasite replication during *in vitro *culture. Although attempts have been made to automate the process of counting malaria blood smears, these approaches have limited utility due to their dependence on high quality smears at a specific cell density. Furthermore, the presence of leukocytes and reticulocytes confounds automated methods as these cell types are frequently misclassified as parasites [1-5]. In addition, the accuracy of automated methods at extremely low levels of parasitemia, such as those that commonly occur in human infections, is much lower than the level required for monitoring parasite survival due to debris in the culture resulting in false positives. Finally, automated methods have an overhead cost in terms of time required to set up hardware and software that only becomes worthwhile once certain economies of scale are reached. For these reasons, we continue to almost exclusively rely on manual counting for monitoring the growth of our malaria parasite cultures.

Cell counting on a hemocytometer or on smears typically employs a multi-unit hand tally counter. These devices are simple manual counters with one or more registers. A lever corresponds to each register, and a single push results in the addition of one event to the corresponding register. The use of multiple registers allows different cell populations to be counted simultaneously (*e.g. *live and dead, uninfected and infected, different leukocyte subsets).

There are a number of inefficiencies associated with this traditional counting method. First, counting large numbers of cells is slowed by the need to press the lever one time for every event despite the fact that cells are often grouped in easily-counted multiples. Second, counting large numbers of slides results in fatigue and repetitive motion strain. Finally, errors in counting cannot be corrected without resetting the manual tally counter. Due to the large volume of cell counting work we conduct, we wished to develop a system that would address these limitations. A software-based counter deployed on minimal hardware was able to greatly improve the efficiency of cell counting in our laboratory.

## Implementation

The software was written in ActivePerl 5.8 under Windows XP and employs Perl/Tk for the GUI and the Win32::Sound module for audio feedback. The complete source code, a binary Windows executable, and configuration files are available at the VersaCount website at SourceForge [6], and at the DeRisi lab website [7].

## Results and Discussion

**Figure 1:**
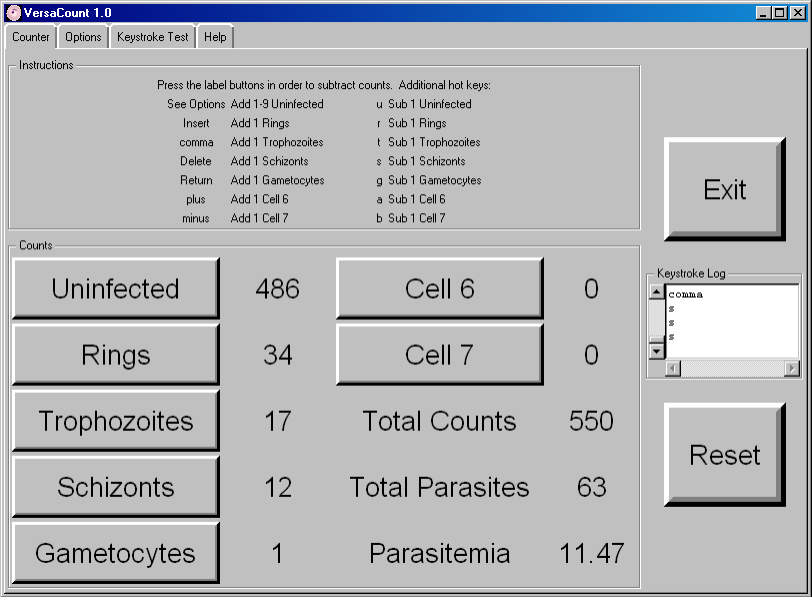
**The VersaCount graphical interface configured for counting the different lifecycle stages of the malaria parasite**.

The VersaCount software incorporates the functionality of a traditional seven-register manual tally counter (Figure 1). In place of input levers, the software was designed to utilize the numeric keypad of a keyboard, which reduces fatigue during extended counting sessions. The software is capable of running on systems with minimal hardware requirements. Our laboratory employs a dedicated counting station outfitted with a FitPC 1.0 (AMD 500 MHz CPU, 256 MB RAM) running Windows XP, a Phylon 8.4" touchscreen LCD, and a Rocketfish wireless numeric keypad. Our system was designed to have a minimal footprint, but any system with similar specifications or better is adequate for use as a cell counting station. The recently popularized category of laptop computers known as "netbooks" would be well suited for the purpose and would cost only a fraction of a hand tally counter with seven registers. If cost is more of a concern than space, older desktop computers which have been decommissioned from general use are another adequate alternative.

### Customizability

The software was designed with a high level of customizability to allow operation with a variety of input devices and counting applications. Although the overall design of the software revolves around the use of a numeric keypad, the designation of keys for the addition and subtraction of events is completely customizable, permitting the use of any keyboard configuration. Additional customization options include the ability to specify names for each register, equations which allow real-time computation of multi-register values, alarm thresholds to alert the user when certain count values have been reached, and sound files that provide distinct audio feedback for each category.

### Equations

Two fields are available for user-specified equations which can be used to compute population frequencies in real-time. This feature allows for rapid, error-free feedback on population counts and frequencies when multiple cell categories are involved. The syntax of the equations is identical to that used in Perl, with each register being tracked by its corresponding variable name ($cell1 through $cell7) and all standard mathematical operations available for use.

### Multi-counting

The most significant efficiency improvement of the VersaCount software over traditional counting methods is the ability to count multiple events with a single key press. The first register (labeled "Uninfected" in Figure 1) is configured as a multi-count category, to which anywhere from one to nine events can be added using a single keystroke. Because the brain is able to process visual clusters of cells much faster than the hand can respond to individual cells, the overall speed of counting on a traditional hand counter is limited by the rate at which the user can repeatedly press a lever or button. With the implementation of the multi-counting feature, the rate limiting step becomes the brain's ability to group and process events. This feature results in substantial time savings when counting hundreds of identical events (up to 30% by our estimates), and becomes especially beneficial when multiple large samples are being counted.

## Conclusions

We have developed software which improves the efficiency of microscopic cell counting. The software greatly enhances productivity when counting large numbers of cells and/or samples, all at a lower cost than most commercially available multi-register hand tally counters.

## Availability and requirements

The Perl source code, Windows executable, and configuration files are freely available at the VersaCount website at SourceForge [6] and the DeRisi lab website [7]. The software is distributed under the terms of the GNU General Public License.

## Competing interests

The authors declare that they have no competing interests.

## Authors' contributions

CCK conceived of the work, coded the software, and wrote the manuscript. JLD participated in designing the hardware configuration, designing the user interface, and writing of the manuscript. Both authors read and approved the final manuscript.
